# Interaction between TNF and BmooMP-Alpha-I, a Zinc Metalloprotease Derived from *Bothrops moojeni* Snake Venom, Promotes Direct Proteolysis of This Cytokine: Molecular Modeling and Docking at a Glance

**DOI:** 10.3390/toxins8070223

**Published:** 2016-07-20

**Authors:** Maraisa Cristina Silva, Tamires Lopes Silva, Murilo Vieira Silva, Caroline Martins Mota, Fernanda Maria Santiago, Kelly Cortes Fonseca, Fábio Oliveira, Tiago Wilson Patriarca Mineo, José Roberto Mineo

**Affiliations:** 1 Institute of Biomedical Sciences, Laboratory of Immunoparasitology “Dr. Mario Endsfeldz Camargo”, Federal University of Uberlândia, Av. Pará 1720, Uberlândia 38400-902, Brazil; maraisacris@doutorado.ufu.br (M.C.S.); tlopes_s@yahoo.com.br (T.L.S.); murilo.vieira@ufu.br (M.V.S.); carolinemartinsm@doutorado.ufu.br (C.M.M.); nandasantiago@hotmail.com (F.M.S.); tiago.mineo@ufu.br (T.W.P.M.); 2 Institute of Biomedical Sciences, Laboratory of Biophysics, Federal University of Uberlândia, Av. Pará 1720, Uberlândia 38400-902, Brazil; kellyfonseca2003@yahoo.com.br (K.C.F.); foliveira@umuarama.ufu.br (F.O.)

**Keywords:** BmooMP-alpha-I, zinc metalloprotease, TNF, TACE

## Abstract

Tumor necrosis factor (TNF) is a major cytokine in inflammatory processes and its deregulation plays a pivotal role in several diseases. Here, we report that a zinc metalloprotease extracted from *Bothrops moojeni* venom (BmooMP-alpha-I) inhibits TNF directly by promoting its degradation. This inhibition was demonstrated by both in vitro and in vivo assays, using known TLR ligands. These findings are supported by molecular docking results, which reveal interaction between BmooMP-alpha-I and TNF. The major cluster of interaction between BmooMP-alpha-I and TNF was confirmed by the structural alignment presenting Ligand Root Mean Square Deviation LRMS = 1.05 Å and Interactive Root Mean Square Deviation IRMS = 1.01 Å, this result being compatible with an accurate complex. Additionally, we demonstrated that the effect of this metalloprotease on TNF is independent of cell cytotoxicity and it does not affect other TLR-triggered cytokines, such as IL-12. Together, these results indicate that this zinc metalloprotease is a potential tool to be further investigated for the treatment of inflammatory disorders involving TNF deregulation.

## 1. Introduction

TNF is a classical pro-inflammatory cytokine involved in the modulation of acute inflammatory responses and host defense mechanisms [1]. However, increased levels of this cytokine are closely associated with degenerative diseases, such as sepsis, rheumatoid arthritis, and inflammatory bowel disease, among others [2]. It is mainly produced by monocytes and secreted as a transmembrane protein (mTNF-26 kDa) and cleaved by the TNF-converting enzyme (TACE), a zinc metalloprotease, in its soluble form (sTNF-17 kDa). Both fragments are biologically active and bind as trimers to either TNF receptors, TNFR1 (also referred as TNFRSF1A, p55, or CD120a) or TNFR2 (also called TNFRSF1B, p65, or CD120b) [3,4]. Currently, monoclonal antibodies against TNF are commercially available to treat TNF-mediated pathologies. These antibodies are most frequently applied for treatment of rheumatoid arthritis, and promising results have been obtained for treatment of other inflammatory disorders [5].

Metalloproteases are enzymes characterized by presenting a catalytic zinc ion in its active site [6]. Snake venom metalloproteases (SVMPs) represent at least 30% of the toxin composition of many viperid snake venoms and they are responsible for hemorrhage through disturbances in the blood coagulation cascade of prey and snakebite victims [6]. However, certain SVMPs lack hemorrhagic activity (P-I class of SVMPs), but present other biological effects, such as inhibition of platelet aggregation, induction of apoptosis, and pro- or anti-inflammatory activities [7,8,9]. SVMPs are phylogenetically most closely related to the mammalian ADAM (a disintegrin and metalloprotease) and ADAMTS (ADAM with thrombospondin type-1 motif) family of proteins and, together, they constitute the M12B clan of metalloendopeptidases [6].

The SVMPs are divided into three main classes according to their domain organization: SVMPs of the class PI contain only the metalloprotease domain in the mature protein, including the canonical zinc-binding motif HEXXHXXGXXH followed by a Met-turn motif. The class PII is comprised of enzymes containing a disintegrin domain following the metalloprotease domain. Class PIII SVMPs contain the metalloprotease domain, the disintegrin-like (Dis-like) domain and a cysteine-rich domain (Cys-rich). Post-translational processing of precursors of some PIII metalloproteinases results in the release of the Dis-like and Cys-rich domains (DC fragment) [10,11]. Additional heterogeneity of these enzymes arises from the occurrence of PII and PIII SVMP dimers, and there are PIII SVMPs that contain an additional subunit constituted by a C-type lectin-like protein, linked to the main proteinase chain by disulfide bonds [7,10,11].

BmooMP-alpha-I isolated from *B. moojeni* snake venom is a fibrin(ogen)olytic and non-hemorrhagic zinc metalloprotease of the class PI SVMPs with a molecular mass of 24.5 kDa [12,13]. This enzyme exerts its biological activity by cleaving first the A-alpha-chain of fibrinogen, followed by its B-beta-chain, but with no effects on the gamma-chain. Also, it lacks hemorrhagic and thrombin-like activities [12].

Previous study of the crystal structure of BmooMP-alpha-I showed that the enzyme presents a catalytic zinc ion displaying an unusual octahedral coordination, which includes three canonical histidines [13]. From this structural study, as well as from comparative sequence analysis, it was concluded that the motif comprising amino acid segments 153–164 and 167–176 adjacent to the methionine-turn is a relevant feature that differentiates non-hemorrhagic and hemorrhagic class P-I SVMPs, and could directly be involved in the development of the hemorrhagic activity [13].

Studies of BmooMP-alpha-I to date have focused only on its fibrin(ogen)olytic and non-hemorrhagic activity. The major aim of the present study was to investigate whether this metalloprotease could modulate TNF inflammatory properties, considering that the precursor form of this cytokine is targeted by TACE, another metalloprotease from the same class (zinc-dependent metalloendopeptidases).

## 2. Results and Discussion

Venoms secreted by snakes constitute a complex mixture of molecules with various biological activities directed to different targets [14]. This is an evolutive adaptation and well-integrated system of proteins and organic constituents, used as a defense by the snakes, as it leads to the immobilization, death, and digestion of the preys [15]. The most evident activity of venoms produced by *Bothrops* snakes is proteolysis, which is responsible for the main clinical manifestations of bothropic acidents [16].

**Figure 1:**
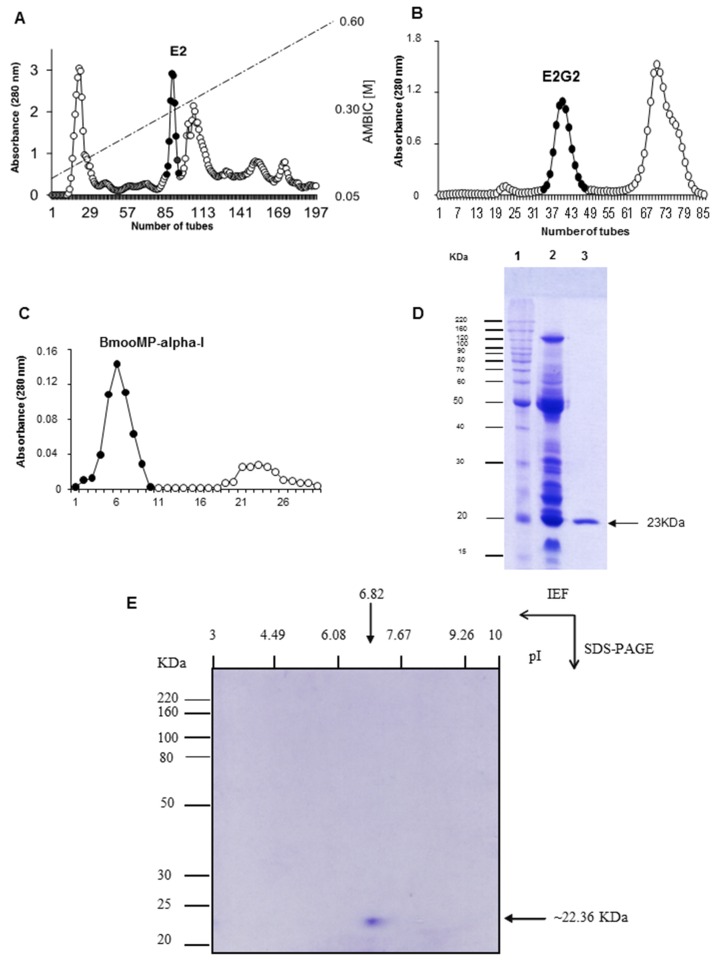
Purification of BmooMP-alpha-I from *Bothrops moojeni* snake venom. (**A**) Separation on DEAE-Sephacel: crude venom (400 mg) was applied on the column (1.7 × 15 cm) and elution was carried out at 20 mL/h flow rate with ammonium bicarbonate (AMBIC) gradient buffer, pH 7.8, from 50 mM to 0.60 M; (**B**) Separation on Sephadex G-75: the active fraction (E2) was applied on the column (1.0 × 100 cm) and elutiom with 50 mM ammonium bicarbonate buffer at pH 7.8 was achieved at a flow rate of 20 mL/h; (**C**) Separation on Benzamidine Sepharose the fraction concentrate (E2G2) was applied on the column (20 × 15 cm) and elution was carried out at 40 mL/h flow rate with 50 mM glycine at pH 3.0. Pooled fractions are indicated by the closed circle; (**D**) SDS-PAGE in 12% (*w*/*v*). Lanes: 1–standard proteins; 2–non-reduced crude venom *B. moojeni*; 3–non-reduced BmooMP-alpha-I; (**E**) 2D electrophoresis of BmooMP-alpha-I solubilized in isoelectric focalization (IEF) solution were resolved by IEF capillary gel and then in 12% SDS-PAGE.

In the present study BmooMP-alpha-I was isolated from crude venom by using combined chromatographic protocols. Ion exchange chromatography on DEAE-Sephacel column resulted in the separation of five protein fractions, (peaks E1–E5) (Figure 1A). Fraction E2, which showed substantial proteolytic activity towards azocasein and fibrinogen [12], was chosen for additional procedure, based on chromatography in a Sephadex G-75 column. These procedures resulted in three peaks named E2G1, E2G2, and E2G3 (Figure 1B). The peak E2G2 showed major protein concentration and proteolytic activity and was submitted for further fractionation based on a Benzamidine-Sepharose column, resulting in two new fractions, named B1–B2. The peak B1 corresponded to the metalloprotease BmooMP-alpha-I (Figure 1C). BmooMP-alpha-I represented a quantity of 8.71% of the whole crude venom of *B. moojeni*, a significant amount if compared with other fibrin(ogen)olytic enzymes isolated from similar preparations [12,15,16,17].

Next, 1D and 2D electrophoretic analysis was carried out of the B1 fraction under non-reducing conditions. 1D SDS-PAGE confirmed BmooMP-alpha-I as a monomer, with apparent molecular mass of 23 kDa (Figure 1D). The BmooMP-alpha-I fraction was further analyzed by 2D SDS-PAGE and the apparent molecular mass was calculated as 22.36 kDa, with pI ~6.82 (Figure 1E).

**Figure 2:**
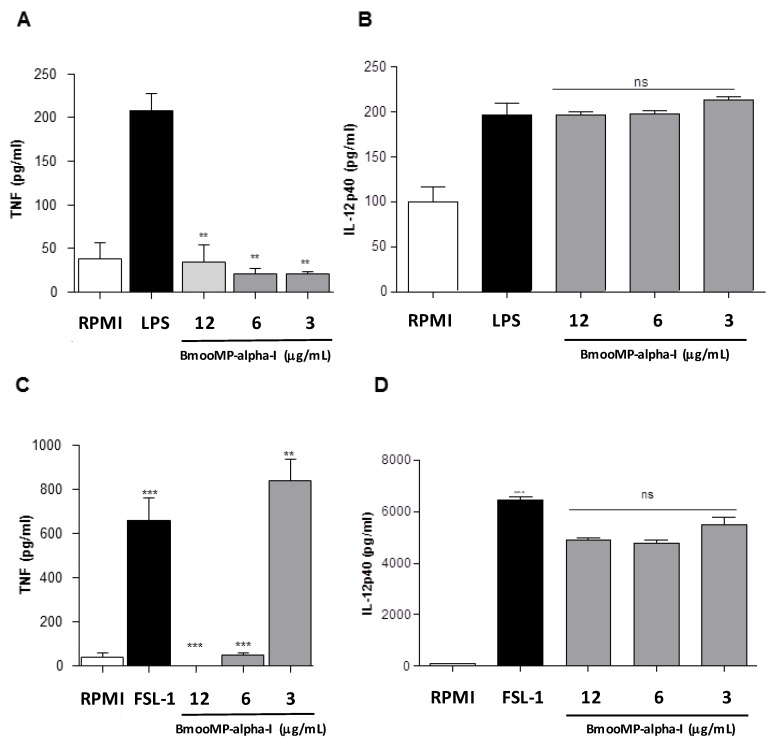
In vitro model for assessment of TNF production. (**A**) Levels of TNF determined after preincubation with LPS and treated BmooMP-alpha-I; (**B**) Levels of IL-12 p40 determined after preincubation with LPS and treated BmooMP-alpha-I; (**C**) Levels of TNF determined after preincubation with FSL-1 and treated BmooMP-alpha-I; (**D**) Levels of IL-12 p40 determined after preincubation with LPS and treated BmooMP-alpha-I. Macrophages were cultured in 96-well plates and after 24 h they were activated with Toll-like receptor (TLR) agonists: LPS (1 µg/mL); FSL-1 (1 µg/mL) or maintained with RPMI medium (control) at 37 °C and 5% CO_2_. Cells were then treated with BmooMP-alpha-I (12 to 3.0 µg/mL) or maintained with RPMI medium (control) for additional 24 h at 37 °C and 5% CO_2_. Levels of TNF and IL-12 were determined by ELISA kit according to the manufacturer’s instructions. Results are expressed as mean ± SD and compared to untreated controls by using Two-way Anova and Bonferroni multiple comparison post-test. ** *p* <0.01 and *** *p* <0.001; ns: no significant in relation to controls (RPMI medium).

The effect of BmooMP-alpha-I fraction was assessed for TNF production by BMDMs stimulated with known TLR ligands. Treatment of BMDMs with BmooMP-alpha-I reduced significantly the TNF detection in LPS-primed macrophages for all enzyme concentrations that were tested (Figure 2A). In contrast, no significant alteration was observed in the levels of IL-12 after treatment with the same concentrations of the metalloprotease (Figure 2B). The priming of BMDMs with TLR2/TLR6 agonist FSL-1 induced strong production of TNF, which could be inhibited by the BmooMP-alpha-I, when tested in concentrations of 12 and 6 µg/mL (Figure 2C). BmooMP-alpha-I was also not able to alter IL-12 production (Figure 2D). Additionally, we observed that this induced effect is independent of cell cytotoxicicity, as determined by MTT cell viability assay using macrophages treated with agonists and/or BmooMP-alpha-I (Figure S1).

**Figure 3:**
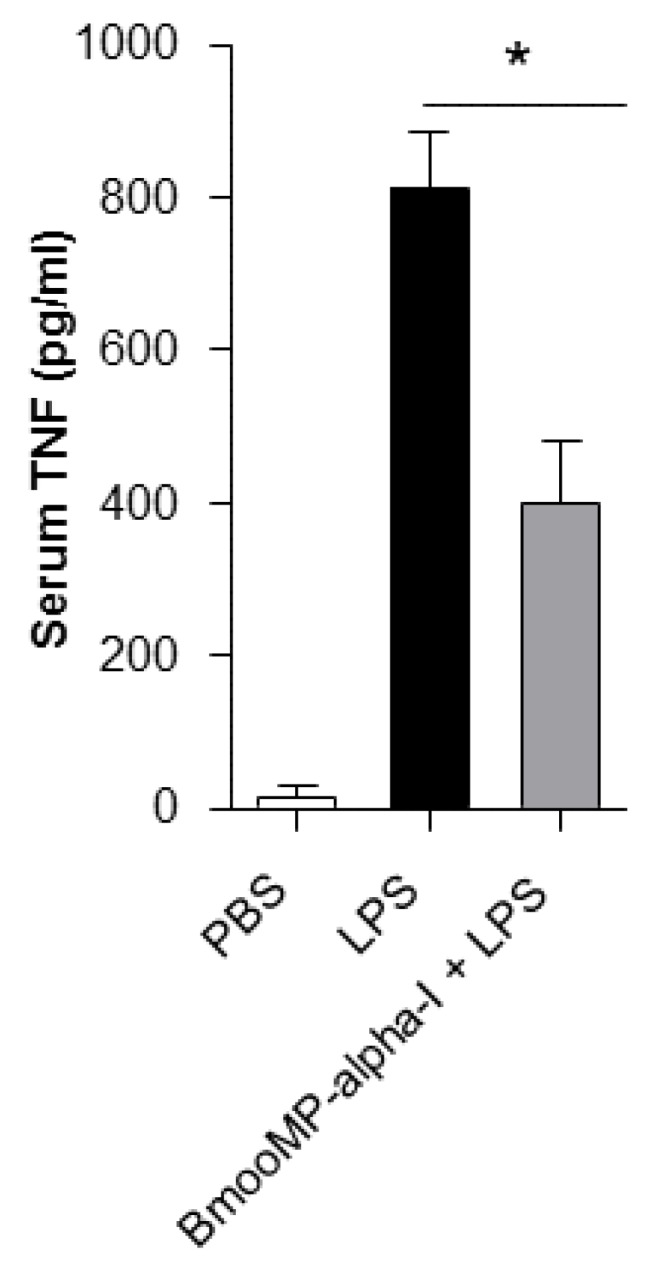
In vivo model for assessment of TNF production. Mice were divided in groups (*n* = 10 per group) treated with the following conditions: PBS (control); PBS plus 100 µg of LPS (PBS + LPS); or BmooMP-alpha-I (50 µg) plus 100 µg of LPS (BmooMP-alpha-I + LPS). All animals received a final volume of 1 mL into their peritoneal cavity; the pretreatment was realized over 1 h and stimulated with LPS for 90 min. Serum TNF levels were measured by ELISA. The results are expressed as mean ± SD in triplicate for each experimental condition, and compared by using One-Way Anova and Tukey’s Multiple Comparison Test. * *p* <0.05 in relation to the group of control (mice that received injection with PBS, only).

To further confirm the inhibitory effect of the BmooMP-alpha-I metalloproteasee on TNF, we examined the effect of this enzyme in a model of LPS-induced sepsis in mice, as it was already known that death caused by septic shock is crucially dependent on TNF production [18]. It was found that the serum levels of TNF were reduced by nearly 50% in animals pre-treated with BmooMP-alpha-I, when compared with mice inoculated with PBS only (Figure 3). LPS is a major structural component of Gram-negative bacteria cell walls and it is able to induce the systemic inflammation observed in septic shock by interacting with TLR4. Upon activation of TLR4, a sequence of signal transduction events occurs, leading to the nuclear translocation of NF-kB transcription factor, which results in transcription of various inflammatory cytokines, such as TNF, IL-1-beta, and IL-12p70. It has been described that these cytokines are responsible for the systemic inflammatory response observed during septic shock [19]. Thus, strategies to decrease the TNF levels could be beneficial to control several pro-inflammatory pathologies, as the blockage of this cytokine improves the prognosis of these diseases [20].

**Figure 4:**
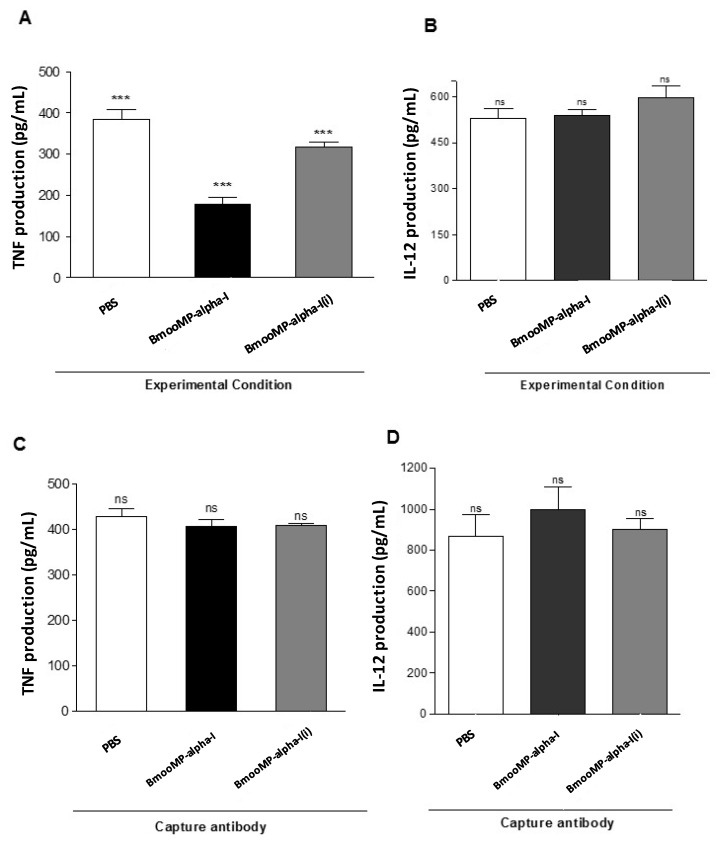
Inhibitory effect of BmooMP-alpha-I on the TNF detection. (**A**) Determination of TNF levels; (**B**) Determination of IL-12 levels. The cytokines were detected after preincubation of capture antibody with 12 μg/mL BmooMP-alpha-I or 10 mM EDTA treated BmooMP-alpha-I (BmooMP-alpha-I(i)) for 45 min at 37 °C; (**C**) Determination of TNF levels (**D**) Determination of IL-12 levels. Now, the cytokines were detected after preincubation of recombinant TNF or IL-12 with 12 μg/mL BmooMP-alpha-I or 10 mM EDTA treated BmooMP-alpha-I (BmooMP-alpha-I(i)) for 45 min at 37 °C. Negative control was incubated only with sterile PBS. Levels of mouse recombinant TNF were quantified by ELISA kit according to the manufacturer’s instructions (DY 410-R & D Systems). Bars represent means ± SD out of five analyses for each experimental condition. Comparisons were carried out by using One-Way Anova and Dunnett’s Multiple Comparison Test. *** *p* <0.001; ns: not significant in relation to the negative controls.

In order to verify whether TNF was directly cleaved by BmooMP-alpha-I, additional experiments were performed. First, the effect of this metalloprotease was determined on degradation of TNF or IL-12 by ELISA. Levels of TNF were determined under different conditions, as TNF and IL-12 standard cytokines or antibodies against them (capture antibodies) were incubated with the following preparations: pure protein (BmooMP-alpha-I); BmooMP-alpha-I inactivated with NA_2_EDTA (BmooMP-alpha-I(i)) It was observed that the levels of TNF decreased by 53.4% after incubation with the pure metalloprotease. In addition, the level of TNF was restored by 80% in the presence of NA_2_EDTA (Figure 4A). In contrast, no significant alterations were observed in IL-12 levels submitted to the same experimental conditions (Figure 4B). As controls, ELISA plates pre-sensitized with capture antibodies were previously incubated with pure protein (BmooMP-alpha-I), or BmooMP-alpha-I inactivated with NA_2_EDTA (BmooMP-alpha-I(i)). As demonstrated in Figure 4C,D, no significant alteration on the levels of TNF was observed. As shown in Figure 4C, the capture antibodies protect TNF from degradation by possibly masking the proteolytic sites in TNF, corroborating with the direct effect of BmooMP-alpha on TNF. Additional evidence was observed on the fact that the amount of TNF was not reduced in the presence of NA_2_EDTA, a chelating agent used to remove bivalent ions, which completely eliminates the biological activity of SVMPs [21]. Furthermore, the polyclonal antibody anti-BmooMP-alpha-I probably binds to different domains from the catalytic domain of the enzyme responsible fot catalyzing the proteolysis of TNF [22,23].

**Figure 5:**
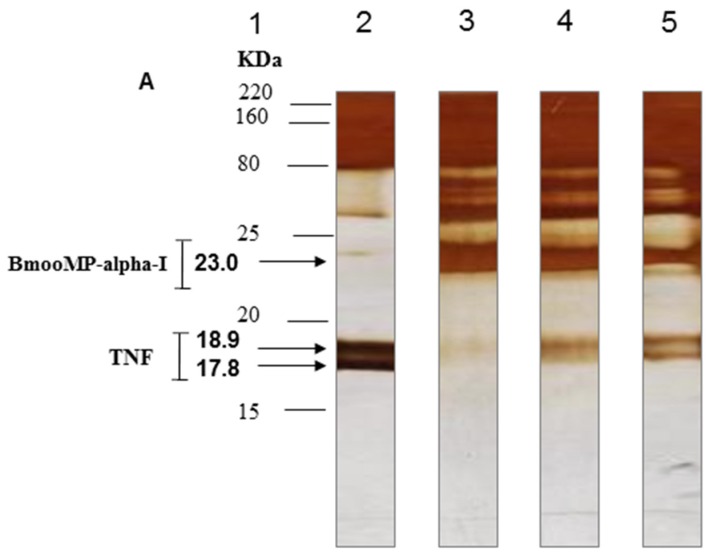

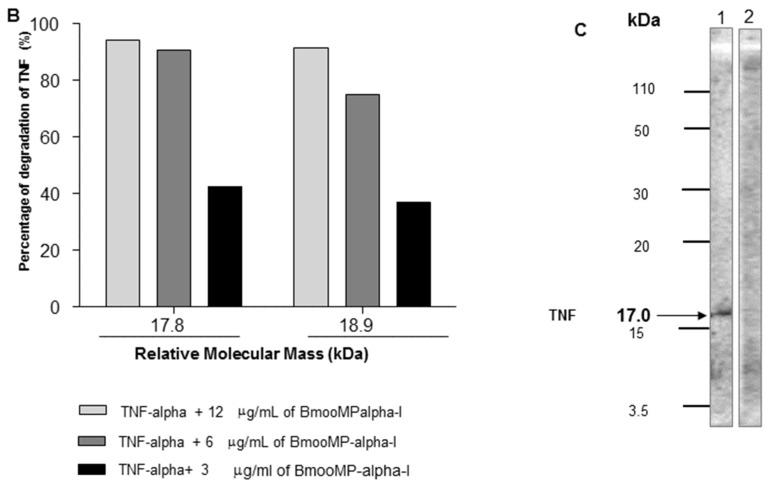
Direct proteolytic effect of BmooMP-alpha-I on TNF cytokine. (**A**) Silver stained 18% SDS-PAGE. Lanes: 1-Molecular weight standard proteins; 2-Mouse recombinant TNF without incubation with BmooMP-alpha-I enzyme (control); 3-4-Mouse recombinant TNF incubated with 12; 6; or 3 μg/mL of BmooMP-alpha-I, respectively, for 45 min at 37 °C; (**B**) Percentage of TNF degradation by BmooMP-alpha-I (12; 6; and 3 μg/mL), determined by band intensity of the reaction products estimated by Kodak 1D image software in relation to negative control versus relative molecular mass of TNF; (**C**) Western blot of TNF protein treated with BmooMP-alpha-I. Immunoreactive bands were developed with ECL (Electron Chemiluminescent) Western substrate and visualized with an enhanced chemiluminescence system. Lanes: 1-Mouse recombinant TNF incubated with PBS (control); 2-Mouse recombinant TNF incubated with 24 µg/mL of BmooMP-alpha-I for 45 min at 37 °C.

To confirm the proteolysis of TNF by metalloprotease BmooMP-alpha-I and to assess whether this effect could present a dose-dependent fashion, additional 1D SDS-PAGE and Western blotting experiments were carried out. The 1D SDS-PAGE with silver stained gel showed that 12 μg/mL of BmooMP-alpha-I was active against TNF and caused a degradation of this substrate, which could be evidenced by the fading of its chains, as demonstrated by the electrophoretic profile of the reaction (Figure 5A). The concentrations of 6 µg/mL and 3 µg/mL of BmooMP-alpha-I impaired the proteolytic activity towards TNF in a dose-dependent fashion, although the effect was still present at the lower concentration (Figure 5A,B). These results obtained by Western blotting demonstrated that BmooMP-alpha-I is able to impair TNF levels to a point that was not recognized by its specific monoclonal antibody (Figure 5C), suggesting that the biological effect imposed by the metalloprotease induced the loss of the TNF tridimensional structure. These findings are in agreement with previous results in the literature concerning a recombinant fibrinogenase rF II, designed from a metalloprotease of *Agkistrodon acutus* snake, which showed a protective role in sepsis by promoting proteolysis of fibrin and TNF, accompanied by a decrease of the plasmatic concentration of this cytokine [24,25]. The same recombinant fibrinogenase rF II showed a protective role in a model of acute severe pancreatitis induced by sodium taurocolate that was dependent of TNF proteolysis [26].

**Figure 6:**
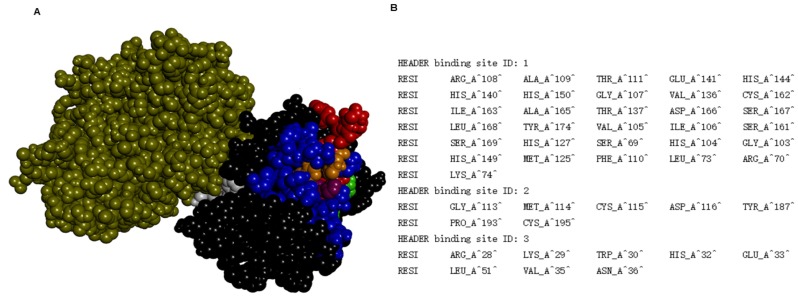
MetaPocket 2.0 Analysis. (**A**) 3D view of the binding cavities in the complex formed between the metalloprotease and TNF. In blue is the binding cavity 1. In green is the binding cavity 2. In white is the binding cavity 3. In purple is the conserved histidine domains. In red is the active site containing the conserved catalytic consense sequence HEXXHXXGXXH; (**B**) Table containing the amino acids sequence of each binding cavity.

To explore the mechanism of the protein-protein interaction, docking analyses of BmooMP-alpha-I protein (3GBO) and TNF (2TNF) were performed using the available protein structures (PDB), followed by structural alignment in the Swiss PDB Viewer. The major cluster of interaction between BmooMP-alpha-I and TNF was confirmed by the structural alignment that presented LRMS = 1.05 Å and IRMS = 1.01 Å (Figure S2). This result is compatible with a realistic complex, once the docking possesses high accuracy had been demonstrated, when (LRMS ≤ 1.0 Å or IRMS ≤ 1.0 Å), intermediate accuracy (LRMS ≤ 5.0 Å or IRMS ≤ 2.0 Å), tolerable accuracy (LRMS ≤ 10.0 Å or IRMS ≤ 4.0 Å), and unrealistic (LRMS > 10.0 Å and IRMS > 4.0 Å) [27,28,29]. The ZDOCK benchmark 3 docking validation, which considers conformational changes occurring between unbound and bound state of the ligand, also presented similar classification to that utilized in the present study, defining the interaction as uncomplicated (C_α_–I_rmsd < 1.5 Å), intermediate (1.5 Å < C_α_–I _rmsd ≤ 2.2 Å), and hard (C_α_–I _rmsd > 2.2 Å) [28,29,30]. Next, we assessed the possible binding cavities and the hydrogen bonds using Swiss Pdb Viewer. It was found that the residues Arg28, His32, Glu33, Val35, Asn36, Ser37, Met38, Gly40, Arg43, Ala49, Asn131, Leu132, Gln133, Glu135, Val136, and Val171 from metalloprotease and Gln31, Arg32, Asn39, Asp42, Leu48, Asp53, Ser86, Tyr87, Glu89, Val91, and Glu 127 from TNF constitute the interactive site of the complex. Additionally, we observed that one of the possible binding cavities interacted with TNF (Figure 6).

**Figure 7:**
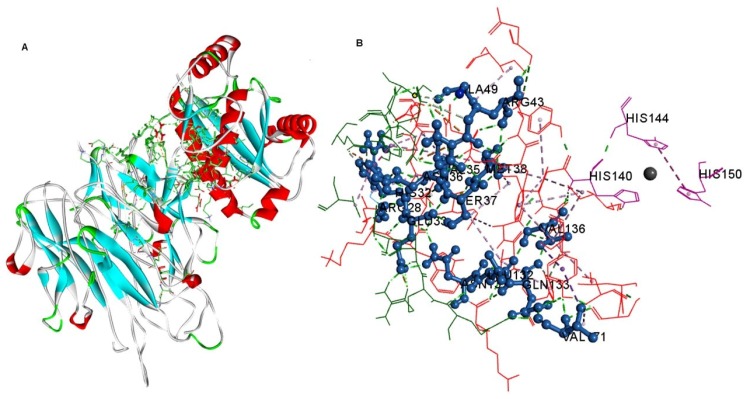
Analysis of the interaction of molecular complexes by Discovery Studio 3.5.0. (**A**) Interaction between the complex residues of 3GBO—2 TNF; (**B**) Demonstration of interacting residues in zoom out view. BmooMP-alpha-I is represented in red, and TNF recombinant murine in green. In purple the Zinc binding site is demonstrated.

In order to evaluate the stability of the potential interactions, an electrostatic analysis was carried out. The interaction between the BmooMP-alpha-I and TNF complex occurs among the following residues, respectively: Glu33-Arg32, Val171-Gln31; Ala49-Asn39; Arg28-Glu89, Arg43-Asp53; Arg43-Glu127; Arg43-Asp53; Arg43-Glu127; Val35-Asn39; Ala49-Asn39; Gln133-Gln31; Gly40-Asp42; His32-Tyr87; His32-Val91; Tyr42-Asn39; Ala49-Ser86; Val35-Tyr87 (Figure 7A,B, Figure S3A–D and Figure S4). This analysis also demonstrated several hydrogen bonds that are important for stabilization of the complex and in promoting the hydrophobic interactions, while being considered the main mechanism of action of metalloproteinases interaction, also indicating a consistent interaction between TNF and BmooMP-alpha-I [31,32]. In addition, it was possible to identify that chains A and C of TNF interact with BmooMP-alpha-I (Figure S2B), and this piece of information was further confirmed by the electrostatic potential analysis (Figure S4).

**Figure 8:**
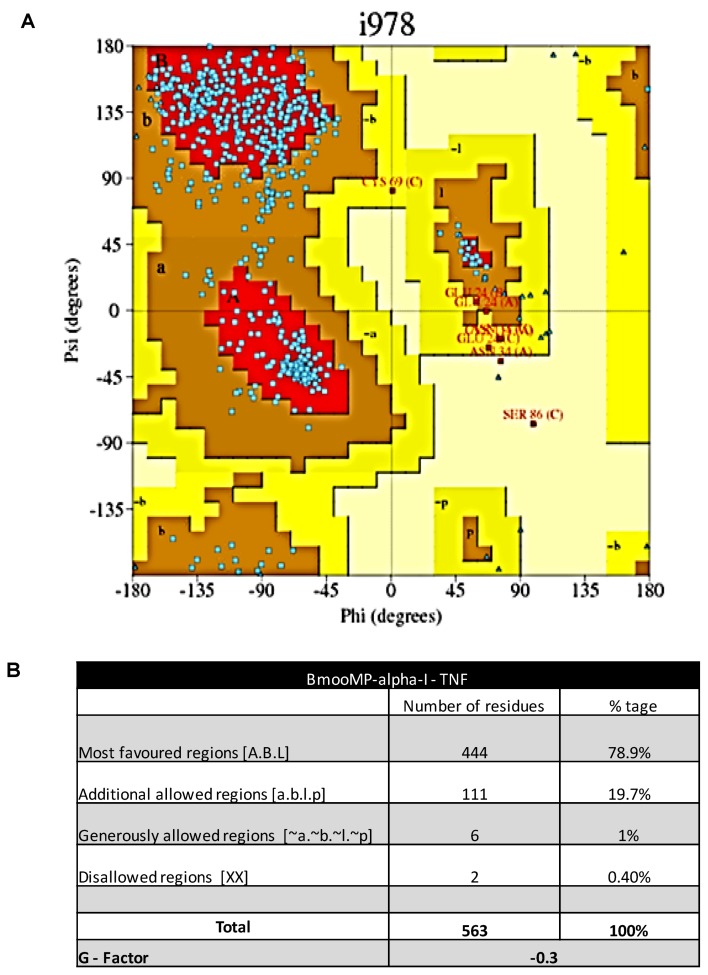
Ramachandran plot: (**A**) The graphic demonstrates the phi-psi torsion angles for all residues in the BmooP-alpha-I and TNF molecular complex. The coloring/shading on the plot represents the allowed phi-psi backbone conformational regions, where the darkest areas (in red) correspond to the most favorable combinations of phi-psi values; (**B**) Details of the residues number in each region of Ramachandran plot and G-score.

The Ramachandran plot and alanine scanning analysis were used to validate the structure of complexes formed by protein interactions. The analysis of the complex in PDB sum generated a Ramachandran plot, where the majority of amino acids residues (*n* = 98%) were located in allowed regions (Figure 8A). Additionally, *G-factor* value (0.43) was determined, which is consistent with a favorable model of interaction (*G-factor* > −0.5) (Figure 8B). Concerning the alanine scanning analyses, the following interacting residues were demonstrated: Arg28, Glu33, His32, Val35, Asn36, Arg43, Val136, Val171 in the metalloprotease and Gln31, Arg32, Asn39, Asp53, Ser86, Tyr87, Glu89, Val91, and Glu127 in TNF. As shown in Figure S5, these residues were considered important for upholding the complex. Therefore, it was found that the following residues are determinant for maintenance of the complex interaction between BmooMP-alpha-I and TNF, respectively: Glu33-Arg32; Val171-Gln31; Arg28-Glu89; Arg43-Asp53; Arg43-Glu127; Arg43-Asp53; Arg43-Glu127; His32-Tyr87; His32-Val91; Ala49-Ser86; and Val35-Tyr87. Indeed, alanine scanning is a powerful method to detect important interactions in protein-protein interfaces, as this detection occurs mainly by the measurement of the effect in the amino acid side-chain change in the Cβ carbon atom on the complex affinity [33,34]. Additionally, individual substitutions of several amino acids with alanine could generate a map that indicates which interactions are critical or not for maintenance of interactive complex. Moreover, alanine scanning is an important tool for identification of hotspot residues in protein-protein interfaces that is essential for complex maintenance [34,35].

The platform CLUSPRO has been generally used for docking analysis, as in actin-actin interaction [36]. This docking application was successfully used in the present study for assess the interaction prediction between BmooMP-alpha-I and TNF molecules. It was observed that the metalloprotease BmooMP-alpha-I interacts with the TNF through the amino acids Arg28, Glu33, His32, Val35, Asn36, Arg43, and Val171. Previous study demonstrated that this zinc metalloprotease (BmooMP-alpha-I) possesses an active site located in the upper domain (about 150 *N*-terminal residues) and lower domain (about 50 *C*-terminal residues), which is consistent with other metzincins, such as adamalysin-II [13]. This active site allows the binding to a variety of residues for different sites located in this protein. In addition, the active site is divided into three major subsites (S2, S1, and S′1 subsites) that can confer certain specificity to BmooMP-alpha-I [36]. In this context, the S1 subsite, which is composed by Ile106, Val136, His140, and Leu168, and the main-chain of residues forming the Met-turn, can interact with large, hydrophobic, neutral, side chains such as Leu, Qln, and Phe [37]. It is important to note that Gln133 interacts with Val 136 which is part of the cleavage subsite S1 of metalloprotease (Figure 7E), corroborating with our hypothesis that TNF is cleaved by this metalloprotease.

**Figure 9:**
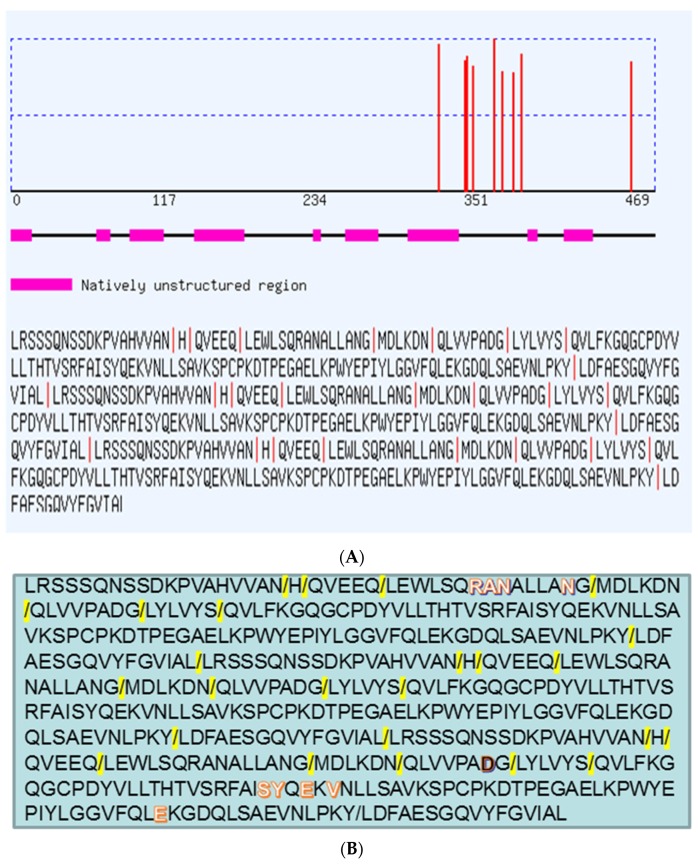
Analysis of the potential cleavage sites in TNF by BmooP-alpha-I metalloprotease using PROSPER by homology with other metalloproteases. (**A**) Predicted cleavage sites P1 in TNF that can be cleaved and the main points of cleavage by metalloproteases that are indicated as a red line in the TNF respective regions; (**B**) Predicted cleavage sites P1 in TNF that can cleaved by metalloprotease in yellow and the predicted amino acids that interact with BmooMP-alpha-I, highlighted in red and white. The most reliable prediction site is highlighted in red and black.

Studies of homology among sequences cleaved by metalloproteases utilizing bioinformatics have been developed once the classes of proteases possess homology on their preferential cleavage sites. For instance, a study pointed out that A and PA-BJ metalloproteases from *Bothropos jararaca* possess propensity for the amino acid arginine in the position 1, although a difference exists in the residue preferred at position 6 and 6′, conferring certain specificity for the class of metalloproteases [38]. The prediction of cleavage sites carried out by PROSPER in the present study demonstrates various points where cleavage can occur, as this metalloprotease owns proline next to the P1 of cleavage (Figure 9). It was observed that several sequences cleaved by this proteinase contain proline [37], corroborating with our results. In addition, the predicted site of cleavage is probably near to the interacting residues, which also indicates the possible cleavage of TNF by this metalloprotease. Furthermore, it is possible to consider that there is recognition of the substrate after conformation change for catalyzing the substrate [39]. In this context, such type of changes in metalloproteases from cavities distant from the active site have been described, indicating a long-range communication network [40,41]. Another important fact is that, although the snake venom metalloproteases and those metalloproteases including Adam 17 (Tumor necrosis factor-α converting enzyme) possess a high conserved domain, located in the regions between the loop connecting H4 an H5 helices, it is necessary to take into account that there are variable regions among metalloproteases. These variabilities are import for substrate recognition, because this feature makes it possible to turn these regions in the substrate-binding pocket wall, as has already been described in the literature [41,42,43]. It is also necessary to consider that the methionine-turns are as important as the zinc catalytic domain for the metalloproteinase activity [40]. Thus, BmooMP-alpha-I may recognize Gln31, Arg32, Asn39, Asp53, Ser86, Tyr87, Glu89, Val91, and Glu127 in TNF and might cleave anteriorly and posteriorly the sequence QLVVPADG, considering that this sequence possesses a proline, which is an important residue recognized by the metalloprotease BmooMP-alpha-I.

It is important to emphasize that the metalloprotease BmooMP-alpha-I is a fibrin(ogen)olytic non-hemorrhagic SVMP that is naturally produced by *B. moojeni* which we found able to hydrolyze TNF. In the literature a high degree of homology among snake venom metalloproteases (SVMPs), metalloproteases from mammalian extracellular matrix (MMPs), and the metalloproteases from disintegrin family (ADAMs), have been described. These families of enzymes are able to degrade subtracts from different sources. In this context, MMPs can cleave cytokines and chemokines, even though the existence of components from the extracellular matrix [44]. Concerning TNF, it is already known that TNF-converting enzyme (TACE) processes the precursor form of this cytokine in order to release its soluble form. Similar to the other members of the ADAM family, the structure of TACE is characterized by distinct domains that include a pro-domain, a metalloprotease, and a disintegrin domain, followed by a cysteine-rich domain containing an epidermal growth factor (EGF)-like repeat, a transmembrane domain, and a cytoplasm tail. Considering the strong evidence that TACE is the major TNF convertase, this enzyme has attracted considerable interest as a specific therapeutic target in several inflammatory disorders, known to benefit from anti-TNF treatment, such as rheumatoid arthritis, Crohn’s disease, and perhaps ulcerative colitis. Consequently, TNF has emerged as an important target for the development of the therapeutic strategies for treatment of chronic autoimmune disorders, and its inhibitors have been approved for clinical use [45]. These studies suggest that the regulation process of the proteolysis from metalloproteases is critical in providing appropriate “*start*” or “*stop*” signaling during an inflammatory response [45]. Thus, an anti-inflammatory agent may be the result of TNF light structural changes, which may hamper its biological activities [46].

In summary, the results described in the present study shed light for future investigations concerning the clinical applications of the zinc metalloprotease BmooMP-alpha-I, since it was demonstrated that this enzyme is able to promote significant proteolysis of TNF. The next step will be to confirm the TNF sequence that is cleaved by this metalloprotease. Therefore, future studies are necessary to determine whether this enzyme could induce protection in experimental models in vivo, particularly those involving TNF as a critical mediator of inflammatory diseases, in order to verify its potential protective role, as well as the existence of possible side effects from its therapeutic use.

## 3. Experimental Section

### 3.1. Animals

Male C57BL/6 mice (18–22 g) were housed in temperature-controlled rooms and received water and food ad libitum until enrolled in experimental conditions. These studies were approved by the Experimental Animals Committee of Universidade Federal de Uberlândia (CEUA-UFU—Protocol # 089/012, approved on 26 September 2012) in accordance with the procedures established by the University Federation for Animal Welfare.

### 3.2. Crude Venom and Toxin

Desiccated *B. moojeni* venom was purchased from Bioagents Serpentarium (Batatais-SP, Brazil). BmooMP-alpha-I was isolated and its purity and biological activity assessed as previously published [12,13], with modifications. Briefly, the purification steps included anion-exchange chromatography on DEAE-Sephacel (Sigma Chem. Co., Saint Louis, MO, USA), followed by size-exclusion chromatography on Sephadex-75 (GE Healthcare, Uppsala, Sweden) and affinity chromatography on Benzamidine-Sepharose (GE Healthcare). The purified toxin was diluted, dialyzed against 50 mM ammonium bicarbonate (pH 7.8), lyophilized, and stored at −20 °C until used. Protein concentration was determined by the Bradford method [47].

### 3.3. Electrophoretic Analysis

Samples of the BmooMP-alpha-I were boiled for 3 min in the presence of sample buffer and resolved in 1D SDS-PAGE at 12%, as previously described [12]. The slab gels were stained with Coomassie Blue R-250, 0.2% (*w*/*v*) in acetic acid:methanol:water (1:5:5, *v*/*v*) solution. The relative molecular mass of the purified enzyme was estimated by Kodak 1D image analysis software (version 3.5, Eastman Kodak Company, Rochester, NY, USA, 2001). BmooMP-alpha-I was also submitted to 2D electrophoresis, by taking120 μg of samples solubilized in 125 μL of isoelectric focalization (IEF) solution (urea 8 M, bromofenol blue 0.002%, CHAPS 2%) and resolved into IEF capillary gel for 12 h, followed by 12% SDS-PAGE.

### 3.4. In Vitro Model for Assessment of TNF Production

To determine the effect of BmooMP-alpha-I in the TNF molecule, it was used an in vitro model based on bone marrow derived macrophages (BMDM) priming with known Toll-like receptor (TLR) agonists, as previously described [48]. Suspensions of 2 × 10^5^ macrophages/well were maintained in 96-well plates in triplicates, for 24 h, in RPMI 1640 medium (ThermoFisher Scientific, Waltham, MA, USA), supplemented with 10% fetal calf serum (FCS; Cultilab, Campinas, SP, Brazil), at standard mammalian cell culture conditions (37 °C and 5% CO_2_). BMDMs were then primed with known TLR agonists (TLR4: LPS from *E. coli* K12, 1 μg/mL; TLR2/TLR6: FSL-1, 1 μg/mL; InvivoGen, San Diego, CA, USA) for 3 h. Next, BmooMP-alpha-I preparations were added at concentrations of 12 µg/mL, 6 µg/mL or 3 µg/mL and the plates were incubated for an additional 24 h. Supernatants were collected and stored at −80 °C until assayed for cytokine production. As negative controls, cells were incubated with medium only, while positive controls consisted of cells incubated with TLR agonists. Cell viability rates were determined by MTT assay, as previously described [49].

### 3.5. In Vivo Model for Assessment of TNF Production

Lipopolysaccharide (LPS) from *Escherichia coli* (serotype O111:B4; Sigma, St. Louis, IL, USA) was injected intraperitoneally (i.p.) in mice to induce acute systemic TNF, following a model for induction of endotoxic shock, as published elsewhere [18]. Briefly, C57BL/6 mice (*n* = 10/group) were pre-treated i.p. with PBS (control) or BmooMP-alpha-I (50 μg), 60 min prior to LPS stimulation (100 µg/mouse). Blood samples were collected from the retro-orbital plexus after 90 min of LPS challenge and serum samples were obtained by centrifugation (500 *g*, 10 min, 4 °C), and stored at −80 °C until assayed for cytokine measurement.

### 3.6. Cytokine Measurements

TNF levels were determined in supernatants from cell cultures and serum samples using a commercial ELISA kit, following the manufacturer’s instructions (R & D Systems, Minneapolis, MN, USA). In addition, IL-12p40 production was also assessed in these samples, using an appropriate kit (BD, Franklin Lakes, NJ, USA). The cytokine concentrations in the samples were calculated by comparison with standard curves of the respective murine recombinant cytokine.

### 3.7. Inhibitory Effect of BmooMP-Alpha-I on TNF Detection

To determine whether BmooMP-alpha-I was able to inhibit TNF detection, mouse recombinant protein (rTNF, 1000 pg/mL; R & D Systems) was pre-incubated with BmooMP-alpha-I (12 μg) or medium alone, as negative controls. Samples of BmooMP-alpha-I inactivated by 10 mM NA_2_EDTA or BmooMP-alpha-I incubated for 2 h at 37 °C with specific polyclonal antibodies (pAb) were included in each assay, as additional controls. In parallel, anti-TNF antibody samples were also submitted to the same conditions, to ensure assay specificity. After incubation, the samples were submitted for detection of TNF by sandwich ELISA (ELISA kit R & D Systems).

### 3.8. SDS-PAGE to Assess Proteolytic Effect of BmooMP-Alpha-I on TNF Protein

Different BmooMP-alpha-I masses (12 μg, 6 µg, and 3 µg) were mixed with rTNF (0.7 ng) in PBS pH 7.2 and incubated at 37 °C for 45 min. Negative controls consisted of metalloprotease masses incubated with sterile PBS only. After incubation, each sample was analyzed by 1D SDS-PAGE (18%), as previously described [50]. Gels were stained by silver nitrate staining kit (ThermoFisher Scientific, Waltham, MA USA) and band intensities were estimated by a dedicated imaging and analysis system (GE Healthcare).

### 3.9. Western Blotting for TNF

TNF proteolysis was further assessed by Western blotting. For this purpose, samples of rTNF (1.4 ng) were incubated with BmooMP-alpha-I (24 μg) or sterile PBS at 37 °C for 45 min. After incubation, the samples were electrotransferred to nitrocellulose membranes, as described [51]. Blotted membranes were blocked with 5% non-fat skim milk in PBS containing 0.05% Tween 20 (PBS-T), for 2 h at room temperature. The membranes were washed with PBS-T and probed with a monoclonal antibody directed to TNF (BD Biosciences, San Jose, CA, USA), diluted 1:250 in 1% PBS-T plus 1% skim milk, for 18 h at 4 °C. Immunoreactive bands were visualized and analyzed after assay development with chemiluminescent buffer (Promega, Madison, WI, USA), through sequential images captured by a proper imaging system (Bio-Rad, Hercules, CA, USA).

### 3.10. Molecular Docking

The X-ray crystallography or nuclear magnetic resonance (NMR) of the primary sequence was searched in the RCSB Protein Data Bank [52]. Both BmooMP-alpha-I protein (3GBO) and recombinant murine TNF (2TNF) were chosen to be analyzed. The crystals were refined using the platform ModRefiner freely available at [53] This platform was assessed to generate algorithms that were used to build and enhance protein structures utilizing the traces of Cα established by two-step atomic-level of energy minimization. Next, molecular docking analyses were performed using the Cluspro program [54] to verify protein interaction. The BmooMP-alpha-I was selected as receptor and TNF as ligand, without selecting a presumed area of interaction. This docking approach utilizes Fast Fourier Transform (FFT), and it is considered an extensive assessment of simplified energy functions of the protein mutual orientations in discretized 6D space. During the procedure, the center of receptor is at the origin of the coordinated system. Meanwhile, the ligand rotates freely, being evaluated through an assumed level of discretization. Several docked structures were created, and the shape complementarity was used as scoring function. The final scoring was given according to the energy function, which was composed of the summation of the shape complementarity, electrostatic, and desolvation contributions. Additionally, the top ten structures resulting from clustering were aligned using a structural alignment by the Swiss Pdb Viewer program and ranked according to LRMS and IRMS values. The best structure, defined after alignment, was further analyzed by Swiss Pdb Viewer for H bonds identification and Discovery Studio 3.5.0 to confirm previous interaction. The protein binding cavities were identified using MetaPocket 2.0 [55]. The PDB sum platform [56] was utilized to create the Ramachandram plot. The structure was considered reliable whether *G factor* > −0.5. Drugscore PPI 2.2 [57] was assessed for the alanine scanning. Finally, the platform Prosper [58] was employed for prediction of possible cleavage sites. This platform utilized homology from other known sequences to be cleaved by proteases, as a parameter to identify points of cleavage in the sequences that had already been determined.

### 3.11. Statistical Analysis

Statistical analysis of data concerning cytokine concentrations was performed using dedicated software (version 6.0h, GraphPad, La Jolla, CA, USA, 2015), using One-way ANOVA or Two-way ANOVA, followed by post-test comparisons. Values of *p* < 0.05 were considered significant and the results are representatives from at least three independent experiments.

## Supplementary Materials

The following are available online at www.mdpi.com/2072-6651/8/7/223/s1, Figure~S1: Cell viability determined by MTT assay of macrophages treated with PRR agonists and/or BmooMP-alpha-I. (A) Cells were cultured in 96-well plates for 24 h and treated with BmooMP-alpha-I (12 to 1.5 μmL) for additional incubation of 24 h; (B) Cells were cultured in 96-well plates for 24 h, activated for 3 h with agonist of Toll-like receptor (TLR): LPS (1 μmL); (C) Cells were cultured in 96-well plates for 24 h, activated for 3 h with agonist of TLR: FSL-1 (1 μmL). Cells were washed with RPMI, and treated with BmooMP-alpha-I (12 to 1.5 μmL) for additional incubation of 24 h; (D) Cells were cultured in 96-well plates for 24 h and incubated with RPMI medium only (absence of BmooMPalpha-I) or activated for 3 h with TLR agonists: LPS and FSL-1 for additional incubation with medium of 24 h. The negative control cells were incubated with RPMI medium only. The negative control cells were incubated with RPMI medium only. The results are expressed as mean ± SD of the percentage of viable cells compared to control and in (A–C) the results are plotted in a non-linear regression represented by a dose response curve with 95% confidence interval. Figure S2: The docking analysis and interacting residues. (A) Structure interaction resulted from CLUSPRO analysis in which BmooMP-alpha-I is represented in blue and magenta, and murine TNF in green and yellow; (B) BmooMP-alpha-I and murine TNF complex in 3D vision determined by PYMOL. Figure S3: Analysis of the BmooMP-alpha-I and TNF interaction by Discovery Studio 3.5.0. (A–D) Demonstration of interacting residues in zoom out view. BmooMP-alpha-I is represented in red, and murine TNF in green. Figure S5: Interaction between the residues of BmooMP-alpha-I and TNF. Interaction specifications between BmooMP-alpha-I and TNF were obtained by Discovery Studio 3.5.0. The letter in front of the residue indicates the chain of the molecules. Figure S4: Alanine scanning analysis. Representation of the amino acid residues according to their sidechain's contribution to the binding free energy, as given in the color scale. The chain representations of the TNF for each residue contributions are shown in white, whereas the correspondent chains of the BmooMP-alpha-I are shown in magenta. (A) BmooMP-alpha-I and TNF chain A; (B) BmooMP-alpha-I and TNF chain B; (C) BmooMP-alpha-I and TNF chain C; (D) The relevant residues for interaction between TNF and BmooMP-alpha-I in terms of ddGcalc (kcal/mol).

## Abbreviations

The following abbreviations are used in this manuscript:

BmooMP-alpha-I: metalloprotease of class P-I from *B. moojeni* snake venom; rTNF: recombinat tumor necrosis factor; TACE: metalloprotease converser of TNF; TNF-RI, TNF-R1, TNFRSF1A, p55, p60 or CD120a: receptor I of TNF; TNF-RII TNF-R2, TNFRSF1B, p75, p80 or CD120b: receptor II of TNF; ADAM: A desintegrin and metalloproteinase; NA_2_EDTA: acid ethylene-diamino-tetraacetic disodium salt; SFB: fetal calf serum; MTT: thiazolyl blue; DMF: *N*,*N*-dimethyl formamide; EGF: epidermal growth factor.

## References

[1] Osta B., Benedetti G., Miossec P. (2014). Classical and paradoxical effects of TNF-alpha on bone homeostasis. Front. Immunol..

[2] Sabio G., Davis R.J. (2014). TNF and MAP kinase signalling pathways. Semin. Immunol..

[3] Wallach D. (2016). The cybernetics of TNF: Old views and newer ones. Semin. Cell. Dev. Biol..

[4] Ramseyer V., Garvin J.L. (2013). Tumor necrosis factor alpha: Regulation of renal function and blood pressure. Am. J. Physiol. Renal. Physiol..

[5] Xixi M.A., Shengqian X.U. (2013). TNF inhibitor therapy for rheumatoid arthritis. Biomed. Rep..

[6] Takeda S., Takeya H., Iwanaga S. (2012). Snake venom metalloproteinases: Structure, function and relevance to the mammalian ADAM/ADAMTS family proteins. Biochim. Biophys. Acta.

[7] Paes Leme A.F., Sherman N.E., Smalley D.M., Sizukusa L.O., Oliveira A.K., Menezes M.C., Fox J.W., Serrano S.M. (2012). Hemorrhagic activity of HF3, a snake venom metalloproteinase: Insights from the proteomic analysis of mouse skin and blood plasma. J. Proteome Res..

[8] Markland F.S., Swenson S. (2013). Snake venom metalloproteinases. Toxicon.

[9] Calderon L.A., Sobrinho J.C., Zaqueo K.D., de Moura A.A., Grabner A.N., Mazzi M.V., Nomizo S.M.A., Fernandes C.F.C., Zuliani J.P., Carvalho B.A. (2014). Antitumoral activity of snake venom proteins: New trends in cancer therapy. BioMed Res. Int..

[10] Barrett A.J., Rawlings N.D., Salvesen G., Woessner J.F., Rawlings N.D., Salvesen G. (2013). Introduction. Handbook of Proteolytic Enzymes.

[11] Herrera C., Escalante T., Voisin M.B., Rucavado A., Morazá D., Macêdo J.K., Calvete J.J., Sanz L., Nourshargh S., Gutiérrez J.M. (2015). Tissue localization and extracellular matrix degradation by PI, PII and PIII snake venom metalloproteinases: Clues on the mechanisms of venom-induced hemorrhage. PLoS Negl. Trop. Dis..

[12] Bernardes C.P., Santos-Filho N.A., Costa T.R., Gomes M.S.R., Torres F.S., Costa J.O., Borges M.H., Richardsond M., Santos D.M., Pimenta A.M.C. (2008). Isolation and structural characterization of a new Fibrin(ogen)olytic metalloproteinase from *Bothrops moojeni* snake venom. Toxicon.

[13] Akao P.K., Tonoli C.C.C., Navarro M.S., Cintra A.C.O., Neto J.R., Arni R.K., Murakami M.T. (2010). Structural studies of BmooMP-alpha-I, a non-hemorrhagic metalloprotease from *Bothrops moojeni* venom. Toxicon.

[14] Pithayanukul P., Leanpolchareanchai J., Saparpakorn P. (2009). Molecular docking studies and anti-snake venom metalloproteinase activity of Thai mango seed kernel extract. Molecules.

[15] Oliveira F., Rodrigues V.M., Borges M.H., Soares A.M., Hamaguchi A., Giglio J.R., Homsi-Brandeburgo M.I. (1999). Purification and partial characterization of a new proteolytic enzyme from the venom of *Bothrops moojeni* (Caiçaca). Biochem. Mol. Biol. Int..

[16] Serrano S.M., Matos M.F., Mandelbaum F.R., Sampaio C.A. (1993). Basic proteinases from *Bothrops moojeni* (Caissaca) venom-I. Isolation and activity of two serine proteinases, MSP 1 and MSP 2, on synthetic substrates and on platelet aggregation. Toxicon.

[17] Torres F.S., Rates B., Gomes M.T.R., Salas C.E., Pimenta A.M.C., Oliveira F., Santoro M.M., de Lima M.E. (2012). Bmoo FIBMP-I: A new fibrinogenolytic metalloproteinase from *Bothrops moojeni* snake venom. ISRN Toxicol..

[18] Allie N., Alexopoulou L., Quesniaux V.J.F., Fick L., Kranidioti K., Kollias G., Ryffel B., Muazzam J. (2008). Protective role of membrane tumour necrosis factor in the host’s resistance to mycobacterial infection. Immunology.

[19] Shanmugam A., Rajoria S., George L.A., Mittelman A., Suriano R. (2012). Synthetic Toll Like Receptor-4 (TLR-4) agonist peptides as a novel class of adjuvants. PLoS ONE.

[20] Croft M., Duan W., Choi H., Eun S.-Y., Madireddi S., Mehta A. (2012). TNF superfamily in inflammatory disease: Translating basic insights. Trend Immunol..

[21] Markland F.S. (1998). Snake venoms and the hemostatic system. Toxicon.

[22] Cardoso R., Homsi-Brandeburgo M.I., Rodrigues V.M., Santos W.B., Souza G.L., Prudencio C.R., Siquieroli A.C., Goulart L.R. (2009). Peptide mimicking antigenic and immunogenic epitope of neuwiedase from *Bothrops neuwiedi* snake venom. Toxicon.

[23] Harrison R.A., Wusterb W., Theakston R.D.G. (2003). The conserved structure of snake venom toxins confers extensive immunological cross-reactivity to toxin-specific antibody Theakstona. Toxicon.

[24] Wang R., Cai J., Huang Y., Xu D., Sang H., Yan G. (2009). Novel recombinant fibrinogenase of *Agkistrodon acutus* venom protects against LPS-induced DIC. Thromb. Res..

[25] Wang R., Qiu P., Jiang W., Cai X., Ou Y., Su X., Cai J., Chen J., Yin W., Yan G. (2008). Recombinant fibrinogenase from *Agkistrodon acutus* venom protects against sepsis via direct degradation of fibrin and TNF-alpha. Biochem. Pharmacol..

[26] Luo S., Wang R., Jiang W., Lin X., Qiu P., Yan G. (2010). Novel recombinant snake venom metalloprotease from *Agkistrodon acutus* protects against taurocholate-induced severe acute pancreatitis in rats. Biochimie.

[27] Huang S.Y., Zou X. (2008). An iterative knowledge-based scoring function for protein-protein recognition. Proteins.

[28] Trellet M., Melquiond A.S.J., Bonvin A.M. (2013). A unified conformational selection and induced fit approach to protein-peptide docking. PLoS ONE.

[29] Krüger D.M., Garzón J.I., Chacón P., Gohlke H. (2014). Drugscore PPI knowledge-based potentials used as scoring and objective function in protein-protein docking. PLoS ONE.

[30] Hwang H., Pierce B., Mintseris J., Janin J., Weng Z. (2008). Protein-protein docking benchmark version 3.0. Proteins.

[31] Apte S.S., Parks W.C. (2015). Metalloproteinases: A parade of functions in matrix biology and and outlook for the future. Matrix Biol..

[32] Verma R.P., Hansch C. (2007). Matrix metalloproteinases (MMPs): Chemical-biological functions and (Q) SARs. Bioorg. Med. Chem..

[33] Chellapandi P. (2014). Structural, functional and therapeutic aspects of snake venom metalloproteinases. Mini-Rev. Org. Chem..

[34] Anand P., Nagarajan D., Mukherjee S., Chandra N. (2014). ABS-Scan: In silico alanine scanning mutagenesis for binding site residues in protein-ligand complex. F1000Research.

[35] Morris A.L., MacArthur M.W., Hutchinson E.G., Thornton J.M. (1992). Stereochemical quality of protein structure coordinates. Proteins Struct. Funct. Bioinform..

[36] Ünlü A. (2014). Computational prediction of actin-actin interaction. Mol. Biol. Rep..

[37] Okamoto D.N., Kondo M.Y., Oliveira L.C., Honorato R.V., Zanphorlin L.M., Coronado M.A., Araújo M.S., Mottae G., Veroneze C.L., Andrade S.S. (2014). P-I class metalloproteinase from *Bothrops moojeni* venom is a post-proline cleaving peptidase with kininogenase activity: Insights into substrate selectivity and kinetic behavior. Biochim. Biophys. Acta.

[38] Zelanis A., Huesgen P.F., Oliveira A.K., Tashima A.K., Serrano S.M., Overall C.M. (2015). Snake venom serine proteinases specificity mapping by proteomic identification of cleavage sites. J. Proteom..

[39] Gunasekaran K., Ma B.Y., Nussinov R. (2004). Is allostery an intrinsic property of all dynamic proteins?. Proteins Struct. Funct. Bioinform..

[40] Fox J.W., Serrano S.M. (2005). Structural considerations of the snake venom metalloproteinases, key members of the M12 reprolysin family of metalloproteinases. Toxicon.

[41] Udi Y., Fragai M., Grossman M., Mitternacht S., Arad-Yellin R., Calderone V., Melikian M., Toccafondi M., Berezovsky I.N., Luchinat C. (2013). Unraveling hidden regulatory sites in structurally homologous metalloproteases. J. Mol. Biol..

[42] Takeda S., Gopalakrishnakone P., Calvete J.J. (2016). Structure-function relationship of modular domains of P-III class snake venom metalloproteinases. Venom Genomics and Proteomics.

[43] Takeda S. (2016). ADAM and ADAMTS family proteins and snake venom metalloproteinases: A structural overview. Toxins.

[44] Ito A., Mukaiyama A., Itoh Y., Nagase H., Thogersen I.B., Enghild J.J., Sasaguri Y., Mori Y. (1996). Degradation of Interleukin 1-beta by matrix metalloproteinases. J. Biol. Chem..

[45] Sisto M., Lisi S., Lofrument D.D., Frassanito M.A., Cucci L., D’Amore S., Mitolo V., D’Amore M. (2009). Induction of TNF-alpha-converting enzyme-ectodomain shedding by pathogenic autoantibodies. Int. Immunol..

[46] Mohammed F.F., Smookler D.S., Khokh A.R. (2003). Metalloproteinases, inflammation, and rheumatoid arthritis. Ann. Rheum. Dis..

[47] Bradford M.M. (1976). A rapid and sensitive method for the quantitation of microgram quantities of protein utilizing the principle of protein dye binding. Anal. Biochem..

[48] Marim F.M., Silveira T.N., Lima D.S., Zamboni D.S. (2010). A method for generation of bone marrow-derived macrophages from cryopreserved mouse bone marrow cells. PLoS ONE.

[49] Mosmann T. (1983). Rapid colorimetric assay for cellular growth and survival: Application to proliferation and cytotoxicity assays. J. Immunol. Methods.

[50] Laemmli U.K. (1970). Cleavage of structural proteins during the assembly of the head of bacteriophage T4. Nature.

[51] Towbin H., Staehelin T., Gordon J. (1979). Electrophoretic transfer of proteins from polyacrylamide gels to nitrocellulose sheets: Procedure and some applications. Proc. Natl. Acad. Sci. USA.

[52] RCSB Protein Data Bank. www.pdb.org.

[53] ModRefiner. http://zhanglab.ccmb.med.umich.edu/ModRefiner.

[54] Cluspro program. http://cluspro.bu.edu/login.php.

[55] MetaPocket 2.0. http://projects.biotec.tu-dresden.de/metapocket/index.php.

[56] The PDB sum platform. http://www.ebi.ac.uk/pdbsum.

[57] Drugscore PPI 2.2. http://cpclab.uni-duesseldorf.de/dsppi/main.php.

[58] Platform Prosper. https://prosper.erc.monash.edu.au/home.html.

