# Supplementary material for: Interaction between TNF and BmooMP-Alpha-I, a Zinc Metalloprotease Derived from Bothrops moojeni Snake Venom, Promotes Direct Proteolysis of This Cytokine: Molecular Modeling and Docking at a Glance
Source: Toxins (Basel). 2016 Jul 20;8(7):223. doi: 10.3390/toxins8070223 (PMC4963855; doi:10.3390/toxins8070223)
Supplement: Supplementary file 1 [file toxins-08-00223-s001.pdf]

Supplementary Materials: Interaction between TNF and BmooMP-Alpha-I, a Zinc Metalloprotease Derived from *Bothrops moojeni* Snake Venom, Promotes Direct Proteolysis of This Cytokine: Molecular Modeling and Docking at a Glance

Maraisa Cristina Silva, Tamires Lopes Silva, Murilo Vieira Silva, Caroline Martins Mota, Fernanda Maria Santiago, Kelly Cortes Fonseca, Fábio Oliveira, Tiago Wilson Patriarca Mineo and José Roberto Mineo

1. Primary Bone-Marrow-Derived Macrophage (BMDM) Cultures

Macrophages were obtained by differentiation of bone marrow from C57BL/6 mice, by using L929-cell conditioned medium (LCCM), as a source of granulocyte/macrophage colony stimulating factor, as previously described [1]. Cells were resuspended in 10 mL bone marrow differentiation media (R20/30), which is RPMI 1640 supplemented with 20% fetal bovine serum (Gibco, cat. 12657-029, Waltham, MA, USA), 30% LCCM, 100 U/mL penicillin, 100 μg/mL streptomycin, and 2 mM of L-glutamine. Cells were seeded in non-tissue culture treated Optilux Petri dishes (BD Biosciences, San Jose, CA, USA) and incubated at 37 °C in a 5% CO_2 atmosphere. Four days after seeding the cells, an extra 10 mL of fresh R20/30 were added per plate and incubated for an additional 3 days. To obtain the BMDM, the supernatants were removed and the attached cells were washed with 10 mL of sterile PBS. Then, 10 mL of ice-cold PBS were added to each plate and incubated at 4 °C for 10 min. The macrophages were detached by gently pipetting the PBS across the dish. Cells were centrifuged at 200× *g* for 5 min and suspended in 10 mL of BMDM cultivation media (R10/5), which is composed of RPMI 1640, 10% fetal bovine serum, 5% LCCM and 2 mM of L-glutamine. Cells were counted, seeded and cultivated in tissue culture plates for the experimental procedures.

2. Cellular Viability Assay

Murine macrophages treated with BmooMP-alpha-I or PRR agonist were assessed to determine cellular viability, which was evaluated by MTT assay, as previously described [2]. In the first step, 10 μL of thiazolyl blue (MTT) was added at a concentration of 5 mg/mL in medium RPMI supplemented with 10% fetal calf serum (SFB), 4 h before the end of culture of BMDM. After the supernatants were removed, the insoluble purple coloured particles produced by viable cells metabolized MTT were solubilized with 100 μL/well of 10% SDS and 50% *N*,*N*-dimethyl formamide (DMF). After 30 min incubation, the optical densities were determined in plate reader at 570 nm. The results were expressed as the percentage of viable cells compared to controls.

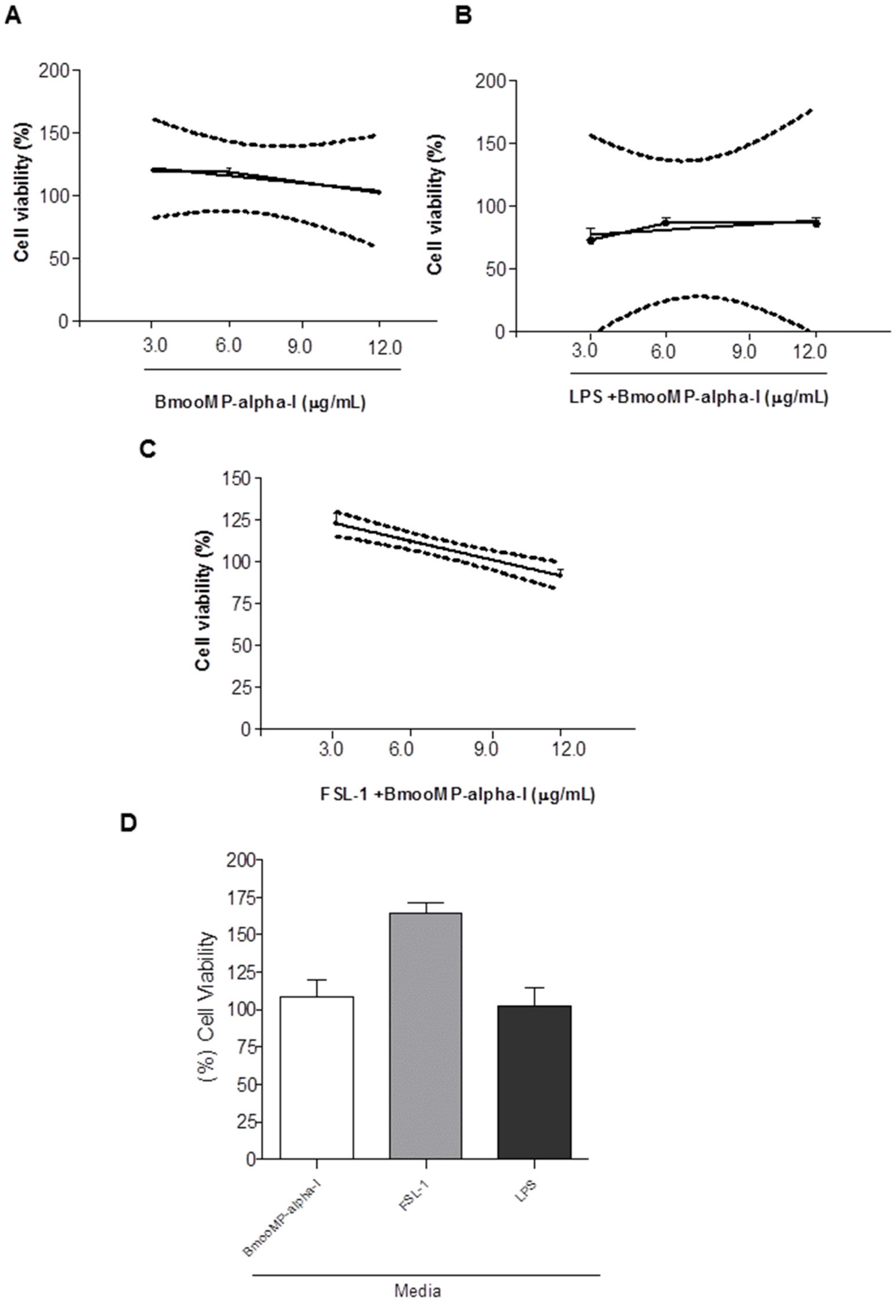

Figure S1. Cell viability determined by MTT assay of macrophages treated with PRR agonists and/or BmooMP-alpha-I. (**A**) Cells were cultured in 96-well plates for 24 h and treated with BmooMP-alpha-I (12 to 1.5 μg/mL) for additional incubation of 24 h; (**B**) Cells were cultured in 96-well plates for 24 h, activated for 3 h with agonist of Toll-like receptor (TLR): LPS (1 μg/mL); (**C**) Cells were cultured in 96-well plates for 24 h, activated for 3 h with agonist of TLR: FSL-1 (1 μg/mL). Cells were washed with RPMI, and treated with BmooMP-alpha-I (12 to 1.5 μg/mL) for additional incubation of 24 h; **D**) Cells were cultured in 96-well plates for 24 h and incubated with RPMI medium only (absence of BmooMP-alpha-I) or activated for 3 h with TLR agonists: LPS and FSL-1 for additional incubation with medium of 24 h. The negative control cells were incubated with RPMI medium only. The negative control cells were incubated with RPMI medium only. The results are expressed as mean ± SD of the percentage of viable cells compared to control and in (**A**–**C**) the results are plotted in a non-linear regression represented by a dose response curve with 95% confidence interval.

A

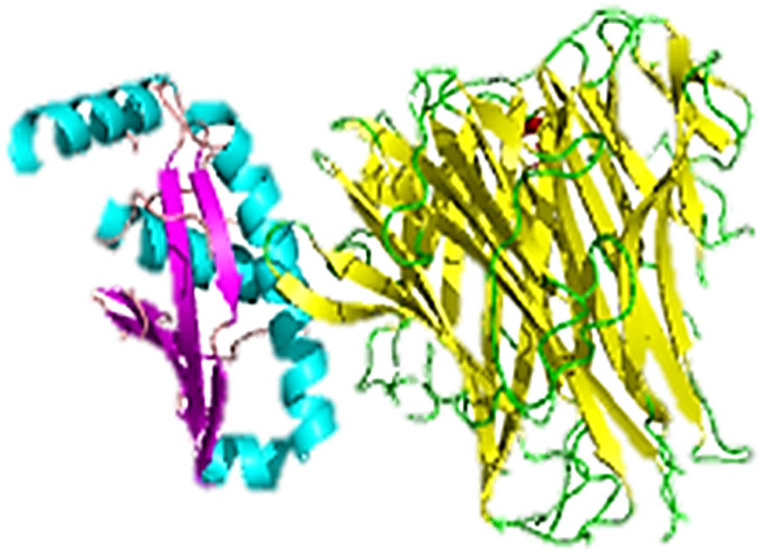

B

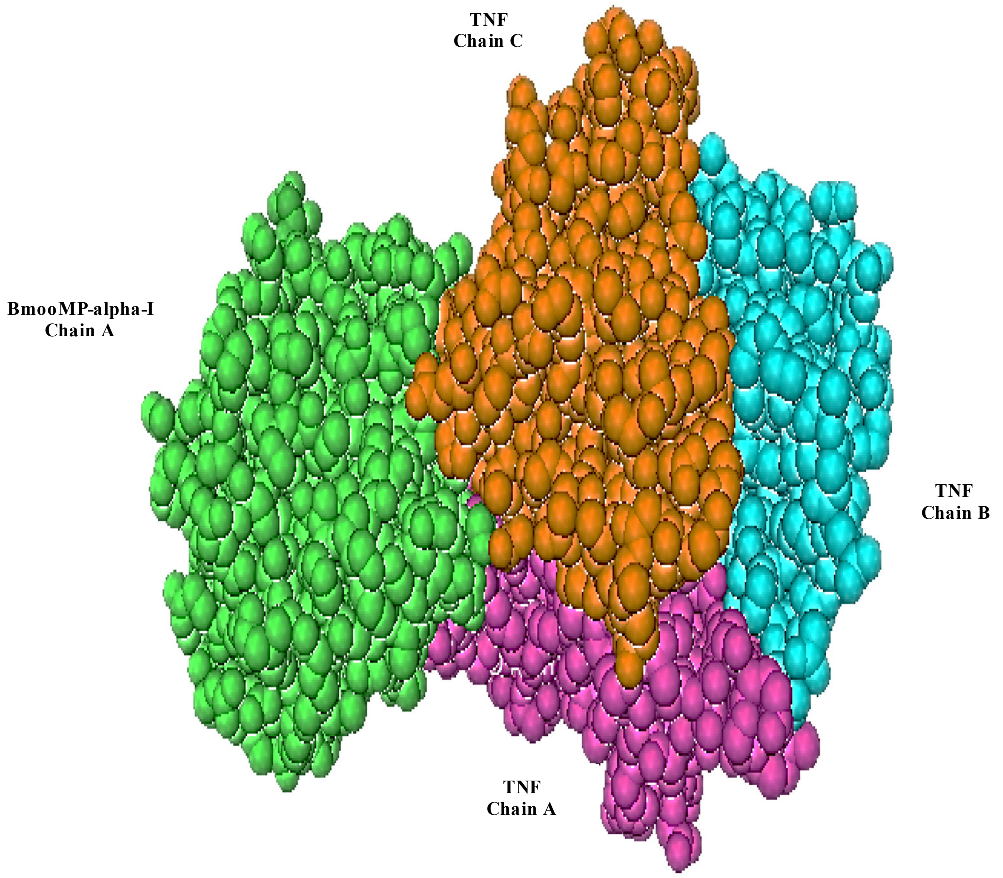

Figure S2. The docking analysis and interacting residues. (**A**) Structure interaction resulted from CLUSPRO analysis in which BmooMP-alpha-I is represented in blue and magenta, and murine TNF in green and yellow; (**B**) BmooMP-alpha-I and murine TNF complex in 3D vision determined by PYMOL.

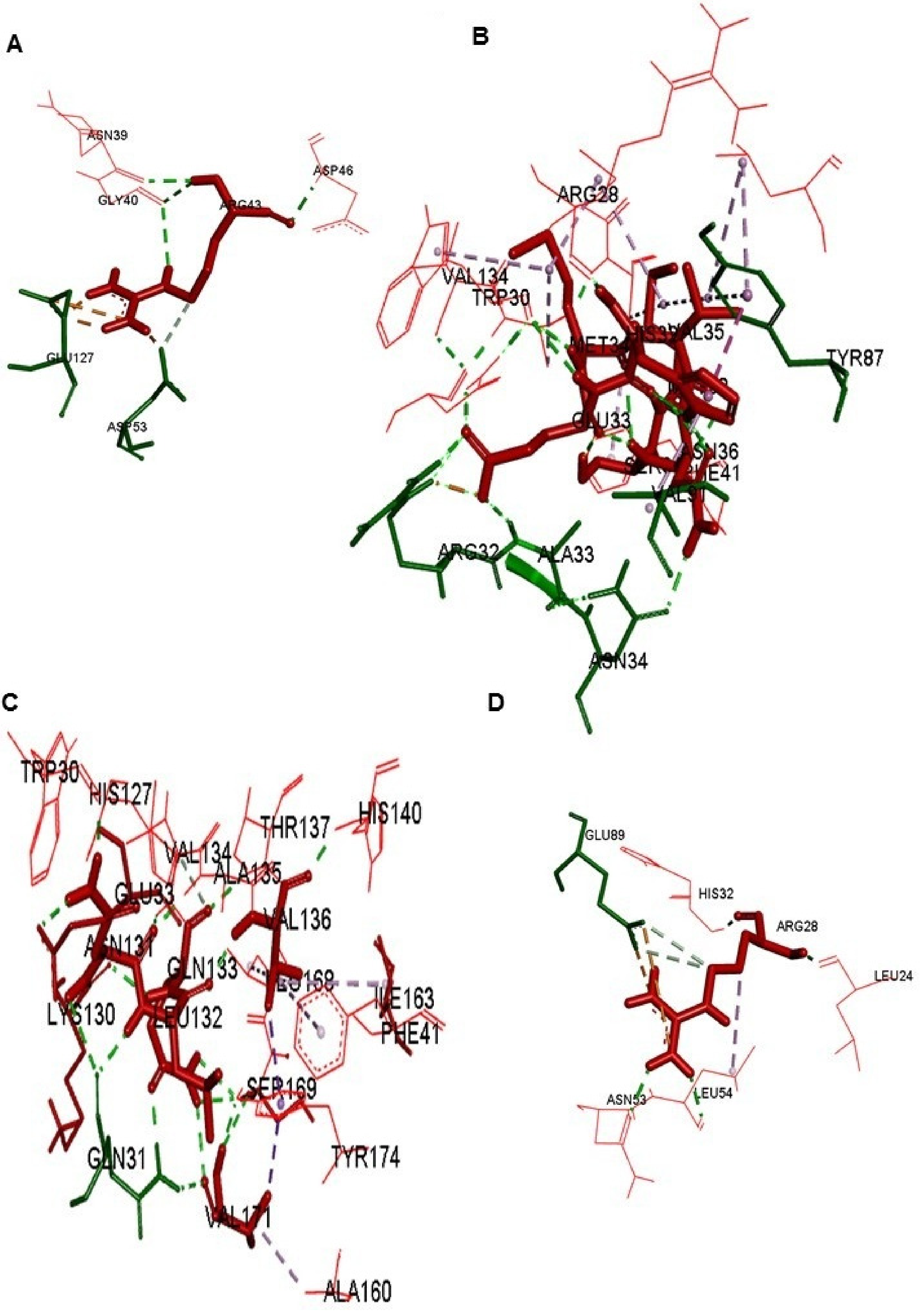

Figure S3. Analysis of the BmooMP-alpha-I and TNF interaction by Discovery Studio 3.5.0. (**A**–**D**) Demonstration of interacting residues in zoom out view. BmooMP-alpha-I is represented in red, and murine TNF in green.

	INTERACTION	DISTANCE	CATEGORY	TYPE	FROM	FROM CHEMISTRY	TO	TO CHEMISTRY
BmooMP-alpha- I and TNF interacting residues	A:ALA49:HN - A:ASN39:OD1	1.95	Hydrogen Bond	Conventional Hydrogen Bond	A:ALA49:HN	H-Donor	A:ASN39:OD1	H-Acceptor
	A:ARG28:CD - C:GLU89:OE1	3.7	Hydrogen Bond	Carbon Hydrogen Bond	A:ARG28:CD	H-Donor	C:GLU89:OE1	H-Acceptor
	A:ARG43:CD - C:ASP53:OD2	3.48	Hydrogen Bond	Carbon Hydrogen Bond	A:ARG43:CD	H-Donor	C:ASP53:OD2	H-Acceptor
	A:ARG43:HE - A:GLY40:O	2.89	Hydrogen Bond	Conventional Hydrogen Bond	A:ARG43:HE	H-Donor	A:GLY40:O	H-Acceptor
	A:ARG43:HH11 - CGLU127:OE2	1.89	Hydrogen Bond;Electrostatic	Salt Bridge;Attractive Charge	A:ARG43:HH11	H-Donor;Positive	C:GLU127:OE2	H-Acceptor;Negative
	A:ARG43:HH12 - C:ASP53:OD2	1.85	Hydrogen Bond;Electrostatic	Salt Bridge;Attractive Charge	A:ARG43:HH12	H-Donor;Positive	C:ASP53:OD2	H-Acceptor;Negative
	A:ARG43:HH21 - C:GLU127:OE2	1.81	Hydrogen Bond;Electrostatic	Salt Bridge;Attractive Charge	A:ARG43:HH21	H-Donor;Positive	C:GLU127:OE2	H-Acceptor;Negative
	A:ASN39:HD21 - A:VAL35:O	1.96	Hydrogen Bond	Conventional hydrogen bond	A:ASN39:HD21	H-Donor	A:VAL35:O	H-Acceptor
	A:ASN39:HD22 - A:ALA49:O	2.09	Hydrogen Bond	Conventional hydrogen bond	A:ASN39:HD22	H-Donor	A:ALA49:O	H-Acceptor
	A:GLN133:HN - A:LYS130:O	2.17	Hydrogen Bond	Conventional hydrogen bond	A:GLN133:HN	H-Donor	A:LYS130:O	H-Acceptor
	A:GLN31:HE21 - A:GLN133:OE1	1.9	Hydrogen Bond	Conventional hydrogen bond	A:GLN31:HE21	H-Donor	A:GLN133:OE1	H-Acceptor
	A:GLY40:O - A:ASP42:N	3.24	Hydrogen Bond	Conventional hydrogen bond	A:GLY40:O	H-Donor	A:ASP42:N	H-Acceptor
	A:HIS32 - C:TYR87	3.56	Hydrophobic	Pi- Pi stacked	A:HIS32	Pi-Orbitals	C:TYR87	Pi-Orbitals
	A:HIS32 - C:VAL91	3.9	Hydrophobic	Pi-Alkyl	A:HIS32	Pi-Orbitals	C:VAL91	Alkyl
	A:TYR42:HN1 - A:ASN39:O	2.31	Hydrogen Bond	Conventional Hydrogen Bond	A:TYR42:HN1	H-Donor	A:ASN39:O	H-Acceptor
	A:VAL136:HN1 - A:LEU132:O	2.34	Hydrogen Bond	Conventional Hydrogen Bond	A:VAL136:HN1	H-Donor	A:LEU132:O	H-Acceptor
	C:SER86:CB - A:ALA49:O	2.96	Hydrogen Bond	Carbon Hydrogen Bond	CSER86:CB	H-Donor	A:ALA49:O	H-Acceptor
	C:SER86:HG - A:ASN39:OD1	4.4	Hydrogen Bond	Pi-Alkyl	C:TYR87	Pi-Orbitals	A:LEU51	Alkyl
	A:ARG32:HE - A:GLU33:OE2	2.00	Hydrogen Bond	Conventional Hydrogen Bond	A:ARG32:HE	H-Donor	A:GLU33:OE2	H-Acceptor
	C:TYR87 - A:VAL35	4.73	Hydrogen Bond	Pi-Alkyl	B:TYR87	Pi-Orbitals	A:VAL35	Alkyl

Figure S4. Interaction between the residues of BmooMP-alpha-I and TNF. Interaction specifications between BmooMP-alpha-I and TNF were obtained by Discovery Studio 3.5.0. The letter in front of the residue indicates the chain of the molecules.

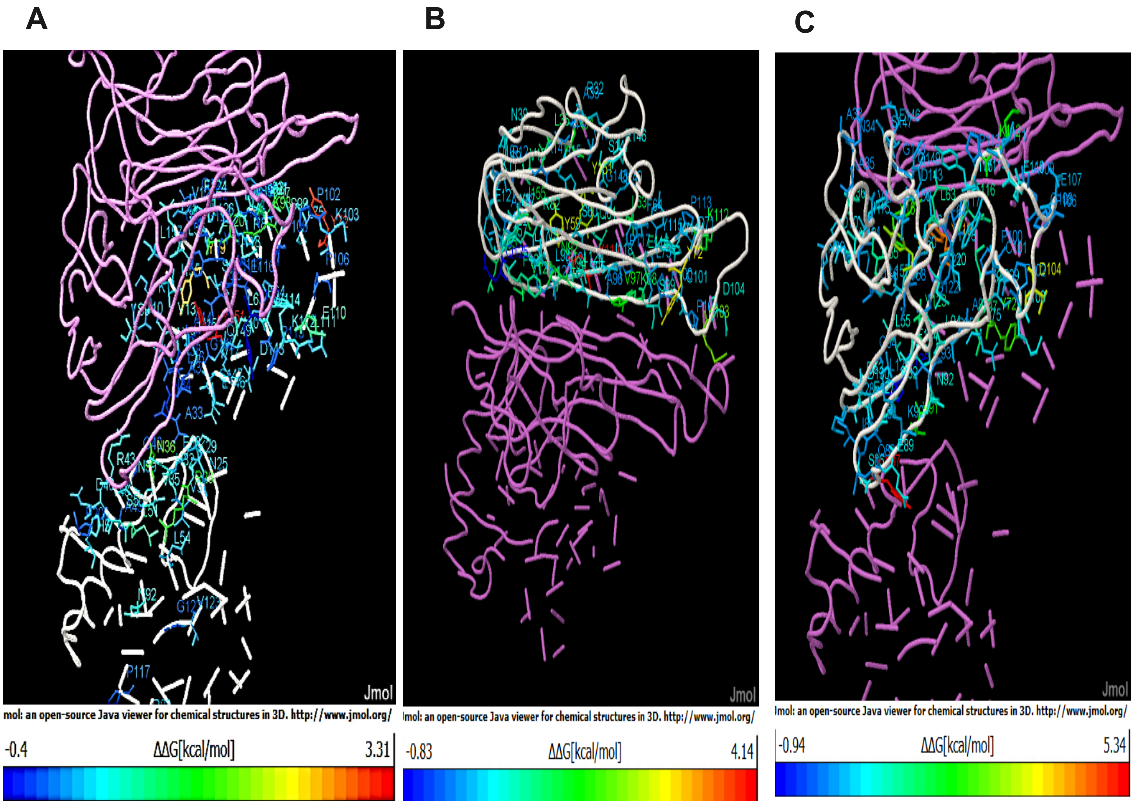

D

	Aa	ddGcalc [kcal/mol]	Buriedness
BmooMP-alpha-I TNF	GLU33	0.28	9.42
	ARG28	1.4	13.69
	HIS32	0.57	17.23
	VAL35	0.48	9.65
	ASN36	2.5	12
	ARG43	0.5	6.8
	ASN39	0.41	14.07
	GLN31	-0.87	14
	ARG32	0.45	15
	ASP53	0.59	5.14
	SER86	0.55	7.74
	TYR87	5.34	5.71
	GLU89	0.12	5.01
	VAL91	2.04	7.67
	GLU127	0.55	9.48

Figure S5. Alanine scanning analysis. Representation of the amino acid residues according to their sidechain's contribution to the binding free energy, as given in the color scale. The chain representations of the TNF for each residue contributions are shown in white, whereas the correspondent chains of the BmooMP-alpha-I are shown in magenta. (**A**) BmooMP-alpha-I and TNF chain A; (**B**) BmooMP-alpha-I and TNF chain B; (**C**) BmooMP-alpha-I and TNF chain C; (**D**) The relevant residues for interaction between TNF and BmooMP-alpha-I in terms of ddGcalc (kcal/mol).

References

1. Marim, F.M.; Silveira, T.N.; Lima, D.S., Jr.; Zamboni, D.S. A method for generation of bone marrow-derived macrophages from cryopreserved mouse bone marrow cells. *PLoS ONE* **2010**, *5*, e15263.
2. Mosmann, T. Rapid colorimetric assay for cellular growth and survival: Application to proliferation and cytotoxicity assay. *J. Immunol. Methods* **1983**, *65*, 55–63.
